# Delayed deep dermal necrosis after jellyfish sting in a 4-year-old female infant

**DOI:** 10.1080/23320885.2018.1533407

**Published:** 2018-10-25

**Authors:** Diana Desax-Willer, Thomas Krebs, Samuel Christen

**Affiliations:** a Children´s Hospital of Eastern Switzerland, St. Gallen/Hospital of St. Gallen, St. Gallen, Switzerland;; b Department for Hand, Plastic and Reconstructive Surgery, Cantonal Hospital of St. Gallen, St. Gallen, Switzerland

**Keywords:** jellyfish sting, children, dermal necrosis, vacuum assisted wound therapy

## Abstract

We report the case of a 4-year-old female infant who developed ongoing deep dermal necrosis of the bilateral legs after jellyfish contact in Thailand. Stepwise radical debridement and vacuum assisted wound therapy seemed to be an effective strategy to prevent progressive soft tissue loss.

## Introduction

Jellyfish stings can result in a variety of symptoms, including pain, swelling, redness, and even severe systemic reactions. The tentacles of some jellyfish species contain undischarged nematocysts and incorporated toxins. Discharge is caused by an array of mechanical and chemical stimuli. It is postulated that immediate or delayed clinical signs are based on toxicological and immunological responses to components of jellyfish venoms and barbed tubules, including proteinaceous porins, neurotoxic peptides and bioactive peptides, collagens, and chitins [1–4].

The emergency treatment of jellyfish stings is based on pain relief, deactivation of the nematocysts and neutralization of the venom. An evidence-based approach does not exist thus far. A variety of topical substances, including acetic acid, water immersion, meat tenderizer, baking soda, or urea, are proposed to inhibit nematocyst discharge and alleviate pain. Birsa et al. reported that some of these substances actually stimulate nematocysts discharge. Nevertheless, they found that the local use of anesthetics (lidocaine) can reduce pain and even prevent discharge of nematocysts [5]. A meta-analysis of randomized, controlled trials based on stings of jellyfish belonging to the genera Physalia, Carukia, and Carybdea jellyfish showed that hot water immersion has a better analgesic effect than the application of ice packs, although there seems to be no benefit of hot water in terms of dermatological outcomes [6]. Cegelon et al. documented the benefit of applying domestic vinegar to inhibit further discharge of nematocysts [7]. An analysis of eight cases of box jellyfish stings in Thailand between 1999 and 2010 underlines the assumed effectiveness of vinegar as a first aid therapy for preventing potentially fatal complications [8].

Only a few case reports of skin necrosis after jellyfish contact can be found in the current literature. Binnetoglu et al. published the case of a 4-year-old Turkish male infant who developed Raynaud’s phenomena after a jellyfish sting, and developed digital necrosis after 10 days. To prevent further progression of necrosis, treatment with intravenous iloprost, steroids, and hyperbaric oxygen was initiated over a period of 4 weeks [9]. A published case from Japan reported digital necrosis of a 31-year-old man after jellyfish contact in Thailand. The treatment consisted of initial fasciotomy, repeated debridement, and subsequently reconstructive surgery [10]. According to the Toxic Jellyfish Network, 3 out of 57 cases of suspected box jellyfish stings in 2008 and in 2009 resulted in dermal necrosis and wound healing disturbance. One patient developed local blisters, swelling, itching, and neuropathic pain without any signs of infection. Another patient with acute wound infection developed local skin necrosis, which required debridement twice; ultimately keloid scars developed. The wound of the third patient became gangrenous, and local debridement was performed [11].

A systematic review of 8 cases (involving 3 children) of box jellyfish stings in Thailand documented 4 fatal and 4 near-fatal courses. In the 4 cases resulting in death, immediate severe pain was followed by systemic signs, such as cardiovascular shock, respiratory failure, and cardiac arrest [8].

We report the case of a 4-year-old female infant who developed ongoing deep dermal necrosis of the bilateral legs after jellyfish contact in Thailand, for which stepwise radical debridement and vacuum assisted wound therapy proved effective.

## Case report

**Figure 1.**
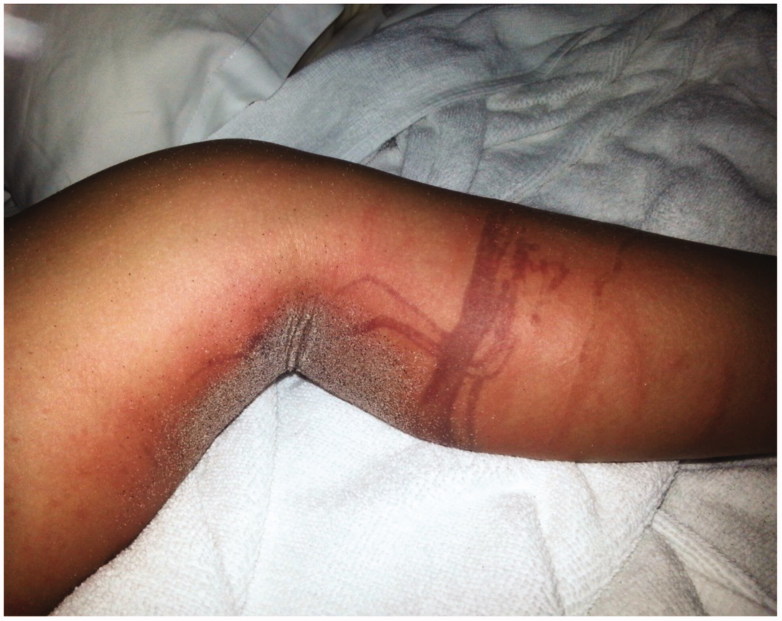
Left leg with dry skin marks 5 days after the accident.

**Figure 2.**
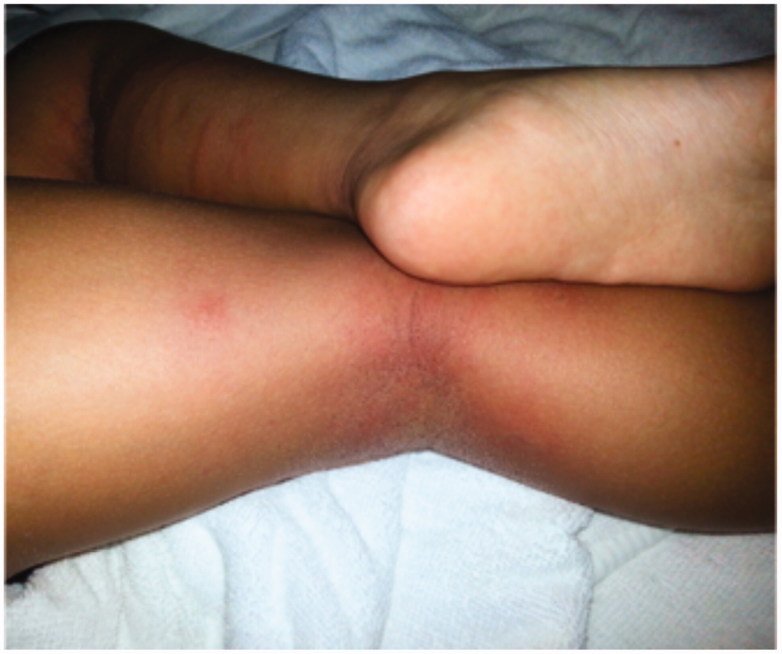
Right poplit with dry skin mark 5 days after the accident.

A 4-year-old female infant, the daughter of a Thai mother and a Swiss father, was stung by a jellyfish during their holidays in Hua Hin, Thailand. The incident occurred at the beach in shallow water. The animal was described to be transparent and had long tentacles (approximately 1 m). The girl immediately experienced intense pain and developed reddish sting marks on her legs. Vinegar was immediately poured over the sting marks. Four hours later, the girl was treated at the local hospital with an unknown topical cream. Five days later, she was presented to the surgical department of a hospital in Bangkok (Figures 1,2). She continued to report pain. According to information provided by the attending doctor, the skin marks were dry, without signs of infection, and there was a mild swelling of the left leg. Systemic therapy with oral prednisone and local treatment with Silvex® cream (containing sulfadiazine and silver) was initiated. The girl developed intense local itching, scabbing occurred, and perifocal redness increased.

**Figure 3.**
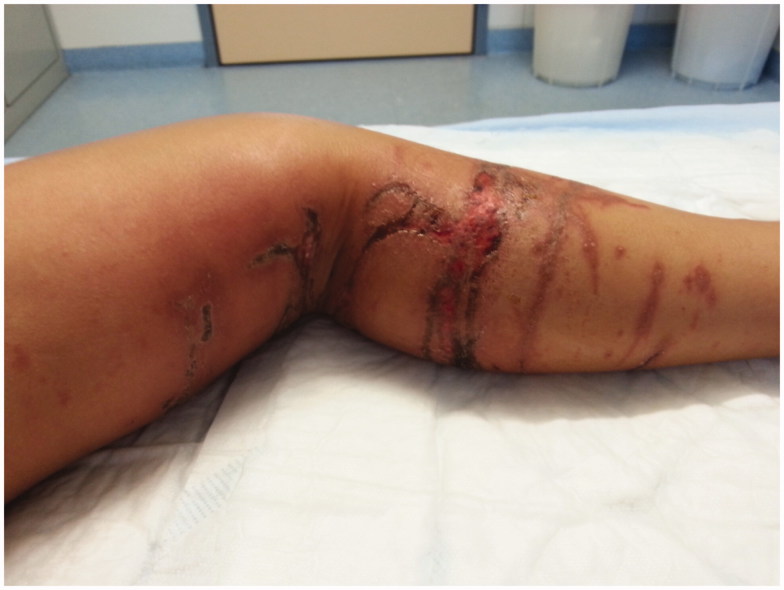
Left leg with superficial dry necrosis with mild perifocal redness 9 days after the injury.

**Figure 4.**
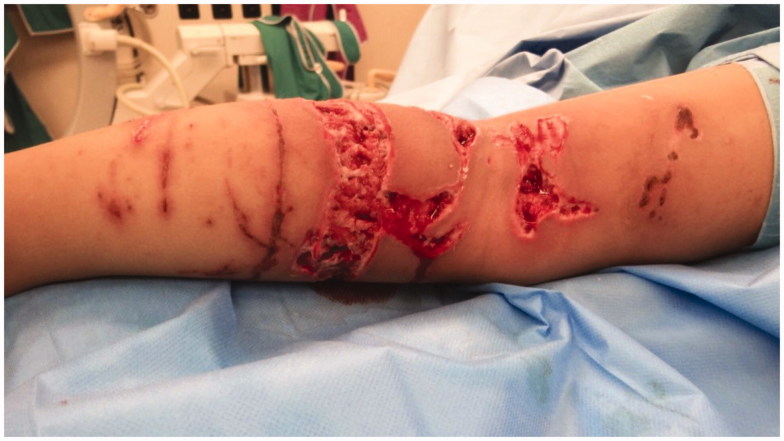
Left leg after radical débridement of the devitalized tissue with very deep, tunnel-like necrosis 14 after trauma.

**Figure 5.**
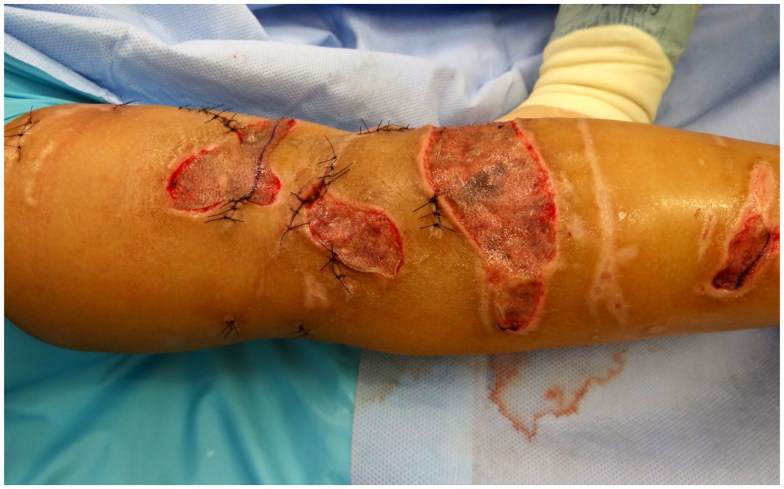
Left leg after closing of all skin lesions by direct wound closure and by split-thickness skin graft.

Nine days after the injury, she presented to our emergency department. She was in poor general condition, with tachycardia and fever. Her vital signs were otherwise stable. Blood markers for inflammation were slightly elevated (c-reactive protein, 65 mg/l). At that point, some of the sting marks of the left leg showed superficial dry necrosis with mild perifocal redness (Figure 3). The few smaller sting marks on the right poplit were dry. Immediate rehydration and analgesic therapy were initiated. After collecting samples for blood cultures, antibiotic therapy with intravenous amoxicillin/clavulanic acid was initiated. Topical dressing with sulfadiazine silver was applied and changed to foam dressing (MepilexAg^®^) the following day. Daily reassessments with dressings and stepwise debridement were performed. At day 14 after the initial event, deep, tunnel-like necrosis was observed on the left leg (Figure 4). Radical debridement of the devitalized tissue under general anesthesia was performed. Vacuum assisted wound therapy (KCI V.A.C.^®^) was initiated. Finally, the ongoing process of deep necrosis resolved and we were able to close all skin lesions of the left leg either by direct wound closure or by split-thickness skin graft (Figure 5). Two weeks after skin grafting, all wounds on the left leg healed.

**Figure 6.**
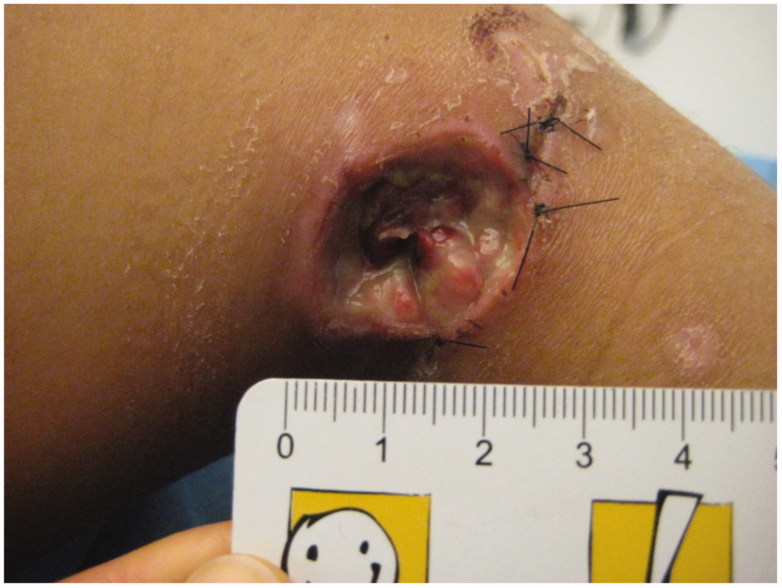
Right poplit with a delayed deep necrosis 19 days after trauma.

Unexpectedly, the initially unimpressive dry skin mark in the right poplit developed delayed deep necrosis 19 days after the initial trauma (Figure 6). The necrotic tissue was excised and vacuum assisted wound therapy was initiated. After eight days, the wound stabilized and we closed the defect with a rotation flap**-**plasty.

**Figure 7.**
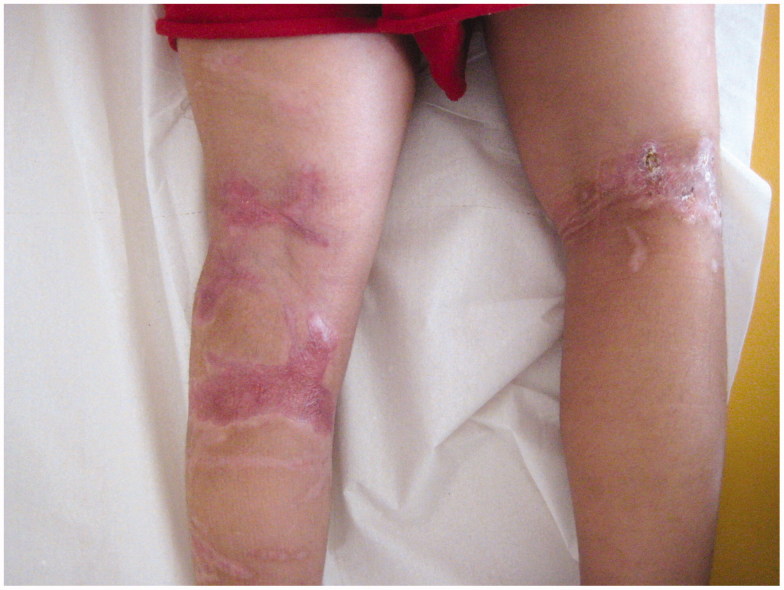
Scars of both legs 8 weeks after the split-thickness skin graft of the left leg.

The scars were initially treated with local massage, and compression stockings were used in combination with massage therapy laters. Figure 7 shows the scars 8 weeks after the split-thickness skin graft of the left leg.

## Discussion

Increased international tourism in recent decades has exposed foreigners to potentially dangerous exotic animals. With growing access to and increased popularity of traveling to subtropical and tropical coastal areas, there has been an increase in severe injuries caused by aquatic animals. During recent years, several new subspecies of highly toxic jellyfish have been reported worldwide.

An increase in the number of box jellyfish in the Gulf of Thailand has been demonstrated in recent years [12].

Jellyfish injuries typically occur in shallow water at the beach. Contrary to adults, children often stay and play in shallow water for a longer time and are therefore at a higher risk of jellyfish stings by animals stranded on the beach, as in our case. Due to their small body surface in relation to the size of the tentacles, children tend to suffer extensive skin lesions. Moreover, in comparison to adult skin, pediatric skin provides less mechanical protection because the epidermis is thinner with less hair coverage.The description of the jelly fish, the skin marks, and the clinical history with intense initial pain and deep delayed soft tissue-necrosis in our case resembles reports of injuries due to Cubozoa subspecies [11–13].

Immunological resistance in the native population, with individuals having a less severe clinical course, has been discussed in the literature. The increased rate of lethal outcomes after jellyfish stings among tourists compared to the local population in Thailand could be a result of hypersensitivity of Caucasians to local jellyfish toxins. However, Thaikurea`s group argued that under-reporting of jellyfish stings exists among the local population [12]. Due to the small number of cases with often incomplete data in the published case series, this hypothesis cannot be proven nor refuted. Nevertheless, the biracial roots of our patient, half Thai and half Caucasian, might be an explanation for the relatively mild and delayed systemic reactions in our case. Furthermore, the affected surface was relatively small and was treated immediately with vinegar, as generally advised in the literature [7,8]. Additionally, the most potent defense cells of a box jellyfish are located at the bottom of the body. According to the marks on the skin, the patient seemed to have contacted only the tentacles. The ongoing process of deep tunnel-like tissue destruction with dry necrosis supports this hypothesis [11]. The delayed soft tissue necrosis could be an immunological reaction to the remaining injected tubules or a reactivation of undischarged nematocysts due to chemical and mechanical stimuli [2]. Our initial debridements of necrotic tissue may not have been sufficiently radical, and may have even stimulated additional nematocysts discharge.

Finally, radical necrosectomy and vacuum assisted wound therapy seemed to stop the progressive soft tissue destruction. Continuous negative pressure was applied to the wound via reticulated polyurethane foam that could be applied on the deep defects. After stabilizing and conditioning the wound base, we could finally reconstruct the skin defect. A meaningful number of studies in children [14–18] shows the efficacy of vacuum assisted wound therapy in difficult wounds. It promotes the preparation of the wound bed by removing excessive wound fluid and infected components and facilitates tissue perfusion and the formation of granulation tissue. Vacuum assisted wound therapy was probably a key component in the treatment of our patient. Apart from necrosectomy, it appears to be effective in removing any remaining components of the tubules.

The wide diversity of jellyfish species with different compositions of toxins and the variety of symptoms makes it almost impossible to define standard therapeutic interventions. Current therapy recommendations are mainly based on case reports, small case series, and single trials with different modes of application and different settings. Hence, the results are hardly comparable and represent low-quality evidence [6].

Primary prevention is the most important intervention. Information, warnings, and beach closures owing to local observations and climatic analysis have been established in many of the affected countries [19,20]. An Australian study showed that jellyfish blooms are predictable. The authors showed that days with prolonged weakening of trade winds are associated with a higher risk of jellyfish stings [21]. Secondary prevention, such as the use of sunscreen lotion with jellyfish sting inhibitors, reduces the symptoms after exposure to jellyfish, as demonstrated in a Norwegian study in 2012 [22].

## Conclusion

Severe and prolonged wound complications can develop after a jellyfish sting. Early radical debridement and vacuum assisted wound therapy seemed to be effective in stopping the potentially progressive tissue destruction induced by jellyfish sting in our case.
